# Overdetection of Breast Cancer

**DOI:** 10.3390/curroncol29060311

**Published:** 2022-05-30

**Authors:** Martin J. Yaffe, James G. Mainprize

**Affiliations:** Physical Sciences Platform, Sunnybrook Research Institute, Toronto, ON M4N3M5, Canada; james.mainprize@sri.utoronto.ca

**Keywords:** breast cancer screening, breast cancer, overdetection, Canadian National Breast Screening Study, overdiagnosis, overtreatment, microsimulation

## Abstract

Overdetection (often referred to as overdiagnosis) of cancer is the detection of disease, such as through a screening program, that would otherwise remain occult through an individual’s life. In the context of screening, this could occur for cancers that were slow growing or indolent, or simply because an unscreened individual would have died from some other cause before the cancer had surfaced clinically. The main harm associated with overdetection is the subsequent overdiagnosis and overtreatment of disease. In this article, the phenomenon is reviewed, the methods of estimation of overdetection are discussed and reasons for variability in such estimates are given, with emphasis on an analysis using Canadian data. Microsimulation modeling is used to illustrate the expected time course of cancer detection that gives rise to overdetection. While overdetection exists, the actual amount is likely to be much lower than the estimate used by the Canadian Task Force on Preventive Health Care. Furthermore, the issue is of greater significance in older rather than younger women due to competing causes of death. The particular challenge associated with in situ breast cancer is considered and possible approaches to avoiding overtreatment are suggested.

## 1. Introduction

The overdiagnosis of breast cancer has been suggested by some to be the largest harm associated with breast cancer screening [1,2,3,4]. Overdiagnosis refers to the diagnosis of breast cancers that normally would not have appeared in a woman’s life, i.e., had not caused harm, before she had died of some other cause. In the case of breast screening, this would come about because of the smaller threshold in lesion size (and presumably development) provided by the screening modality. This provides lead time, and it is this lead time that contributes to the reduction in mortality and morbidity that has been demonstrated in women who participate in screening compared to those who do not [1,5]. If cancers detected earlier through screening would not have surfaced or done harm if they had remained undetected, then they can be considered to have been overdiagnosed, or more correctly, overdetected [6], a term that will be used throughout the remainder of this article when referring to detection by screening, while “overdiagnosis” will be used to refer to pathologic assessments.

**Figure 1:**
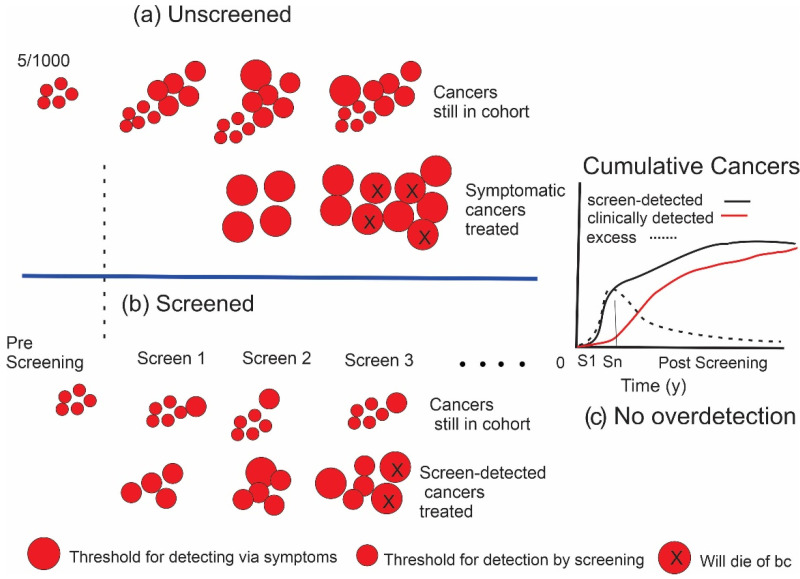
(**a**) Illustrates the initiation and growth of breast cancers in an unscreened population (e.g., 1000 women). Size of the lesion is represented by the diameter of the discs. X indicates cancers that will result in death. (**b**) The effect of screening. In (**a**,**b**), the lower rows indicate cancers that have been detected and treated, while upper rows show cancers in the cohort that have not yet been detected. (**c**) Difference (excess) in the cumulative number of cancers found in screened (black curve) versus unscreened (red curve) individuals, depicted in the graph as the dashed line, increases during the period of screening. In this example where there is no overdetection, cancers are found and treated earlier in the screened group; however, after screening ends at Sn, the number in the unscreened group will catch up over time, eliminating the excess.

Consider a cohort of women of the same age at a given time point. It would be expected that a certain number of breast cancers, illustrated schematically in Figure 1a as discs, would be initiated in the cohort each year. The cancers would vary in size and growth rate according to a variety of driving factors. At an early point, as illustrated on the left in the figure, they would not have yet reached the threshold for detectability; however, as time elapses, the cancers grow and at some point become large or noticeable enough to be detected by the women or by a clinician. Additionally, as shown in the figure, at a point that may occur before or after the threshold for clinical detectability is reached, they have become sufficiently advanced that they will be destined to become lethal (discs indicated with “x”s), or at least their treatment would impose considerable morbidity. The initiation of cancer is a continuous process over time with a rate that is age-dependent, so that as time progresses new, earlier cancers are added to the population of previously undetected cancers that have grown larger.

The principle of screening is illustrated for an identical cohort of women in Figure 1b. If a suitable test is available to which the cohort is exposed at regular intervals and the threshold for lesion detectability is smaller than the clinical detection threshold, then cancers can be found and treated before they reach that clinical threshold. In this screened cohort, the cancers that are detected and treated are shown on the lower track, while those that remain undetected in those women are shown on the upper track. The time that it takes for cancers to grow from the size threshold of the screening test to the threshold for clinical detectability is the lead time afforded by screening. The term “size” is used loosely here as a surrogate for detectability because other factors that develop over time such as changes in morphology may also affect the detection threshold. The expectation is that there would have been less progression in size, a lower probability that metastasis has occurred and a greater chance of avoiding death from those earlier cancers. This paradigm has been demonstrated to be correct through multiple randomized trials, case–control and observational studies, e.g., [1,5,7].

**Figure 2:**
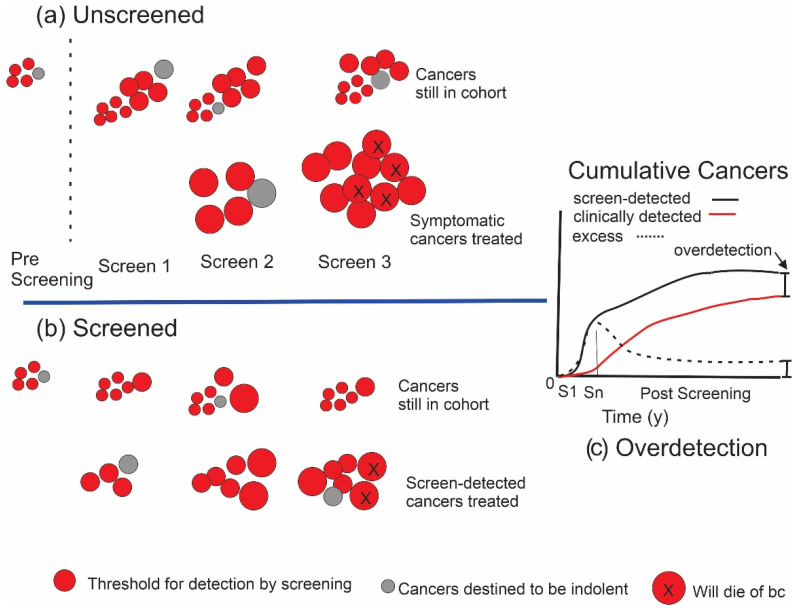
Initiation and growth of breast cancers in the presence of cancers with limited malignant potential. (**a**) An unscreened population. Grey discs indicate cancers that are destined not to be lethal. (**b**) The effect of screening. Lower row indicates cancers that have been detected and treated, while upper row indicates cancers in the cohort that have not yet been detected. (**c**) Overdetection. After screening ends at Sn, the initial excess of cancers in the screened grouped will not be completely eliminated over time.

Eventually, most of the equivalent cancers found earlier in the screened cohort would surface in the unscreened women due to symptoms or accidental detection. In the case of very slow growing (indolent) cancers (grey discs in Figure 2), however, these may not have the potential to progress beyond a certain point or to metastasize and, therefore, would not become lethal or at least not been clinically detectable in the absence of screening before the individual had died of some other cause. Under these conditions, the woman would never have been aware that she had cancer. This phenomenon of overdetection is illustrated in Figure 2b. More of these cancers with limited malignant potential will be detected in a screened population. To the extent that this occurs, the total numbers of cancers detected (and treated) in a cohort of women participating in screening will exceed the corresponding numbers in an unscreened cohort (Figure 2c).

The actual diagnosis of breast cancer is performed by a pathologist on biopsied tissue. A concern regarding overdetection is that a woman who otherwise would not have experienced the anxiety and other negative factors associated with having breast cancer would have become a breast cancer patient.

Overdetected cancers are real cancers and should not be confused with the so-called false positive results of screening, where further imaging or biopsy, triggered by an equivocal screening examination, demonstrates that suspicious results on screening are not cancer. The main point is that there is no direct benefit to the individual from finding overdetected cancers. They are currently an unavoidable collateral finding associated with the earlier detection and treatment of other cancers that would indeed otherwise likely become lethal.

Overdetection by screening can be considered as having two components: (1) detection of nonprogressive cancers and (2) detection of cancers that are progressive, but where the progression is sufficiently slow such that they would not have been detected in unscreened women before they would have died from a cause other than breast cancer. In a recent publication, for women in the age range 50–74 screened biennially who were monitored by the Breast Cancer Surveillance Consortium in the U.S., Ryser et al. estimated the rate of overdetection at 15% [8]. In their Bayesian inference study of 718 cancer diagnoses in 36,000 women, they estimated that one-third of overdetected cancers were indolent, while the other two-thirds were progressive but had not emerged before death had occurred due to another cause. Overdetection via the second mechanism is more likely to occur in older than younger women at time of screening because competing causes of death are higher in the former and, therefore, it is more likely that a cancer will not be detected in her unscreened counterpart before she dies.

Overdiagnosis, in its true sense, occurs when the pathology examination is not able to distinguish potentially aggressive from indolent cancers. The harms of overdiagnosis are the morbidities associated with overtreatment if this occurs. The same limitations can result in underdiagnosis and subsequent undertreatment, with a heightened probability of recurrence or death. Both of these are harms of the diagnostic process and the processes leading to therapeutic choices. It is worth mentioning that not all cancers that are overdetected are overdiagnosed. In some cases, the pathologist can identify disease at a very low risk for recurrence at biopsy. However, unlike the trend toward active surveillance in prostate cancer, where some men choose not to be treated for minimal disease, most women currently receive some level of treatment after a diagnosis of breast cancer. Some of these cancers are undoubtedly overtreated. It is, of course, also possible for cancers detected symptomatically to be over or underdiagnosed.

The main difference between screen-detected and symptomatically detected cancers is that the former tend to be smaller and earlier stage making the diagnostic procedure more challenging. This implies that overdiagnosis is more likely to occur in in situ than in invasive disease. The probability of detecting in situ cancer is greatly increased with screening and, therefore, these lesions require special consideration.

### 1.1. In Situ Cancers

In situ cancers are rarely detected in unscreened women, whereas in a cohort of women routinely screened with mammography, they constitute 20–30% of detected cancers. It has been argued that in situ cancer (which, here, will be loosely referred to as ductal carcinoma in situ or DCIS) should not be considered as a cancer in that, in itself, it does not have the potential to be lethal. If this were the case, then it could be considered that in situ cancer alone might be responsible for an overdetection rate of 20–30%. Certainly, of those cancers overdetected because they are nonprogressive, in situ cancers likely represent a large proportion. Glasiou et al. used Australian registry data to estimate overdetection for various cancers and concluded that for breast cancer there was an overall 22% overdetection of which 9% was for in situ cancers [9].

Nevertheless, in situ disease cannot simply be dismissed as being innocuous. It is well established that, if treated by breast conserving surgery alone, there will be ipsilateral recurrence in about 28% of cases and half of these will appear as invasive cancer [10,11,12]. The use of radiation therapy reduces local recurrence by a factor of two. More recent work by Solin et al. showed recurrence rates of 25% for high grade lesions 1 cm or smaller and 14% for larger (2.5 cm or larger) low or intermediate grade in situ cancer. Again, in each case, about half the recurrences were as invasive cancer [13]. The optimization of strategies of how to manage in situ cancers detected by screening is, therefore, a topic of great interest and some efforts in this direction will be described later in this article.

### 1.2. Estimating the Amount of Overdetection from Screening

There have been many attempts to estimate the amount of overdetection that would result from screening. All of these suffer from various limitations, and this is responsible for wide variation among estimates [5,8,14,15]. For example, Bleyer and Welch [16] extrapolated historical breast cancer incidence data before the onset of breast cancer screening from the SEER Registry to predict what incidence should be in the current era and compared with actual incidence to estimate the excess that they assumed was attributable to overdetection from screening. While conceptually this approach is sound, uncertainties in the year-to-year increase in background age-specific incidence rates, lack of information in SEER on the mode of cancer detection and other sources of variability made their calculation extremely unreliable. Small differences in the assumptions of the values of some of the extrapolation parameters could result in very high estimates of overdetection or even of underdetection [8,17,18]. 

Puliti et al. and Etzioni and Gulatti have identified several of the critical factors required in the estimation of overdetection [19,20] and these include accounting for effects of lead time from screening and differences in cancer risk between comparison groups [19]. Puliti et al. have suggested that when these effects have been appropriately accounted for, the fraction of cancers that have been overdetected is on the order of 1–10%.

Ideally, overdetection would be assessed through a randomized trial, where women in one arm receive screening and those in the other do not. This would eliminate possible differences between the two groups that could be responsible for differences in breast cancer incidence. Both arms would be followed for cancers detected during the period of the screening intervention and for a time period afterwards that is no less than the lead time provided by screening. To avoid bias, it is essential that the quality of the follow-up is identical for the two trial arms. During the post-intervention period, neither group would receive screening. The number of breast cancers occurring in each group would be carefully and thoroughly monitored and the difference would provide a measure of overdetection.

In such a trial, it would be expected that initially there would be an excess of cancers in the screened group due to their earlier detection. After a delay due to the screening lead time, the corresponding cancers would begin to appear in the control group and there would be a compensating decrease in the excess as illustrated in the graph in Figure 1c. If there was no overdetection occurring, then after the appropriate follow-up time, the excess would be neutralized. 

Such an idealized trial is almost impossible to achieve. As in all randomized screening trials, there will be crossover effects due to the noncompliance of women assigned to screening as well as some women in the control group seeking screening outside the trial. If this occurs, it will cause an initial reduction in the measure of overdetection.

The screening behavior of women after the period of the intervention will also affect the estimated overdetection. The measure will be most accurate if neither group receives screening during the post-intervention follow up. Given human behavior this situation is unlikely to be achieved. If there is more post-intervention screening in the screening arm, overdetection will be overestimated and, if there is a greater degree of screening in the control group, the estimated overdetection fraction will be reduced.

### 1.3. Example–Canadian National Breast Screening Study

An example of the problems of estimating overdetection can be seen in the publication by Baines et al. of their revised estimates of overdetection of breast cancer using data from the two randomized controlled trials in the Canadian National Breast Screening Study (CNBSS) [21]. This analysis was an update from Miller et al., 2014, who had originally provided estimates based on the merged data from the two studies [22]. The revised estimates by Baines et al. were considerably larger than the previously published values.

The calculation used by Baines et al. was very simple. In each arm of the RCT, the total number of breast cancers found, which comprised screen-detected cancers or other cancers, were totaled over the period of observation. The estimated overdetection at a given point in time was obtained by dividing the difference in these totals between the study and control groups (the excess cancers) by the number of screen-detected breast cancers in the study group during the period of intervention.

Different authors have employed other denominators in this calculation [1]. Those who wish to accentuate the effect tend to choose the smallest number and vice versa. It is not clear that any particular choice is most correct, but the effect on the estimate of overdetection can be large and comparison of studies requires that the same denominator be used in all cases.

In CNBSS1, women in the age range 40–49 years in the intervention (MP) arm received annual mammography and physical examination, while the control group (UC) received a single physical examination at entry followed by “usual care” in the community, whose nature was undefined [23]. Women aged 50–59 at entry in the intervention (MP) arm of CNBSS2 received mammography plus clinical examination by a nurse (by a physician in Quebec) annually, while those in the control (PE) arm received annual clinical examination only [24].

The estimates of overdetection by Baines et al. are far higher than values published by other authors based on data from randomized trials or observational studies of service screening [19,21]. We, therefore, attempted to examine the results reported by Baines et al. to determine if these estimates were supported by their data. We also conducted microsimulation for the purpose of understanding mechanisms that could lead to the discrepancy in results.

## 2. Materials and Methods

For each of the two studies, CNBSS1 and CNBSS2, Baines et al. reported the cumulative number of invasive cancers and total (invasive and in situ) cancers that had accrued in each trial arm during the period that screening took place (denoted here as Years −4 to 0) and at 1, 2, 3, 4, 5, 10, 15 and 20 years after the study screening examinations terminated [21]. The differences between these two sets of numbers represented the in situ cancers. We observed the patterns in the accumulation of invasive, total and in situ cancers over time to assess if these patterns reflected those expected in a cohort of Canadian women.

### Modelling

To predict the expected number of cancers in a randomized trial of screening, we utilized the OncoSim-Breast model, a microsimulation tool, based partially on the NCI CISNET Wisconsin-Harvard model that has been described previously [25,26]. OncoSim Breast has been validated by comparing its estimates with empirical breast cancer incidence both in an era prior to the implementation of screening programs and after programs were in place. There is no explicit modelling of a mechanism for overdetection in the algorithm; however, a range of cancer progression and growth rates are incorporated in the software. This would provide a distribution of cancers with varying levels of aggressiveness in the simulated population. Some of the slower-growing cancers “created” in the model should exhibit the phenomenon of overdetection. In particular, the model treats in situ and invasive cancers as parallel, partially independent processes. As in situ cancers develop over time, they have a probability of either transitioning into the invasive phenotype or remaining in situ and this can also give rise to overdetection.

The modeling represents a study even more rigorous than the conditions of a randomized trial in that the characteristics of the simulated participants are not only statistically similar, but rather they are perfectly matched; essentially, the simulation creates a study in which each woman in one cohort is matched with her identical twin in the other. For CNBSS1, we simulated a set of birth cohorts of women equally distributed over birth years, such that women would be in the age range of 40–49 at the beginning of the trial. The model inputs assume that prior to the beginning of the trial there is no screening in either arm. For half of the women (MP arm), the model was configured to estimate the number of in situ and invasive breast cancers that would be detected each year if the women received annual screening mammography and clinical examination. For the other half (UC), the model simulated a single screen by clinical examination in the year of entrance to the study and then followed the cohort without any screening intervention, again estimating the number of in situ and invasive cancers that would surface.

For CNBSS2, the same approach was taken; however, the interventions for the two study arms included a total of 40,000 women who would be in their 50s at the onset of the trial. The MP group received clinical examination and mammography annually, while the PE group received only annual clinical breast examination.

For both studies, for the purpose of modelling, it was assumed that no screening occurred in either trial arm after the intervention period. To reduce stochastic noise in modeling, 32 million histories were run and the results were scaled down to the 25,000 women in each arm for CNBSS1 and 20,000 for CNBSS2.

## 3. Results

### 3.1. CNBSS Findings

**Figure 3:**
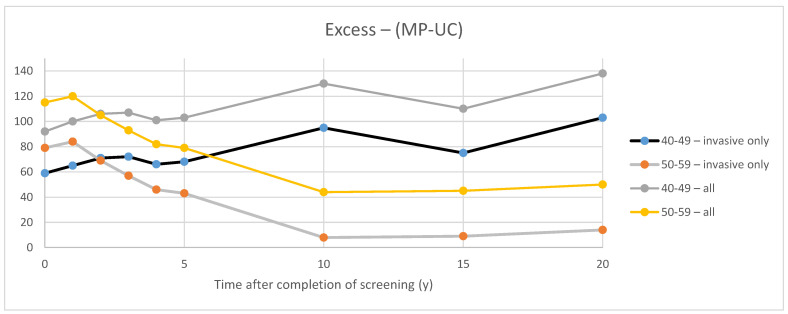
Excess invasive cancers in the study group compared to the controls in the two CNBSS trials.

Figure 3 illustrates the excess in cancers found in the Study arm compared to the Control arm in each of the CNBSS trials. For invasive cancers, at the end of 5 years of the screening intervention (defined here as time 0), there was an excess of 59 and 79 cancers in the younger and older groups, respectively. When all cancers (invasive and in situ) were considered, the corresponding excesses were 92 and 115 cancers, respectively. It can then be inferred that there were 33 excess in situ cancers found in CNBSS1 and 36 in CNBSS2 over the period of screening. However, there is a dramatic difference between the trials in how the excesses varied as time elapsed after the screening intervention in the two trials.

Using the definition employed by Baines et al. for observation periods between t = 0 and 20 years after succession of screening (t = 20 y), for invasive cancers, the estimated overdetection fraction ranged from 28% to 48% at t = 0 and at t = 20 y, respectively, in the women in their 40s and between 29% and 5% for women in their 50s. Estimates were higher (36–53% in the younger group and 35% to 15% at t = 0 and 20 y, respectively, in the older group) when both all cancers (in situ and invasive) were considered.

In CNBSS2, for invasive cancers, the behavior over time is as would be expected with the excess gradually diminishing over the first 10 years after the period of screening as cancers gradually surface (without the benefit of screening lead time) in the control group. When the in situ cancers are included, the absolute excess that they represent at t = 0 appears to persist over time.

In CNBSS1, perplexingly, the excess of invasive cancers actually increases over the 20-year post-screening follow-up period. As in CNBSS2, the absolute contribution of in situ cancers to the excess appears to be essentially constant.

### 3.2. Modelling

**Figure 4:**
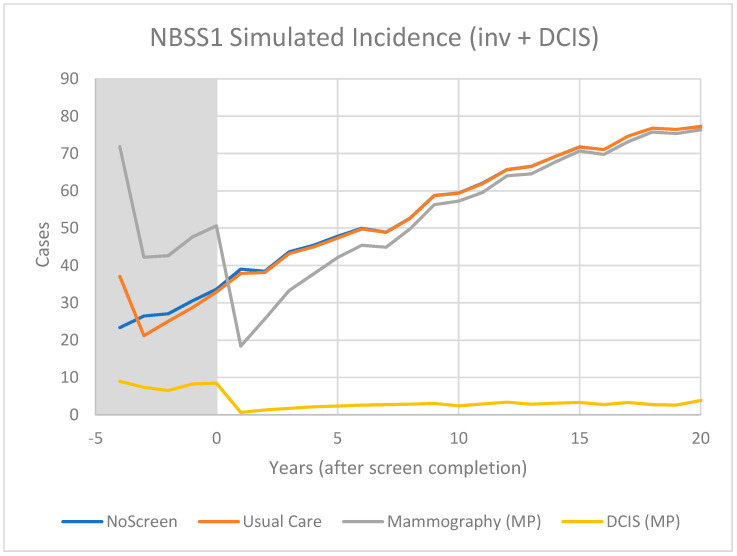
Predicted cases of breast cancer (OncoSim-Breast model) for women who are unscreened and in the Mammography or Usual Care arms of the CNBSS1 trial.

**Figure 5:**
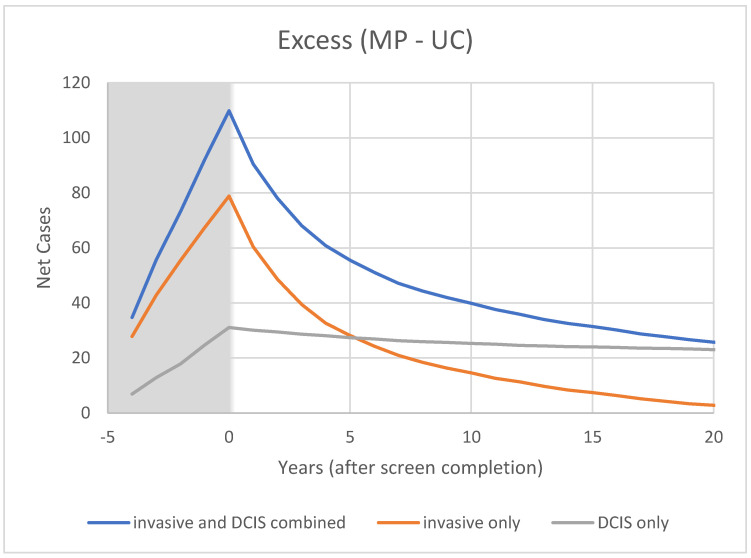
Modeled cumulative excess breast cancer detection in CNBSS1 from OncoSim-Breast. Shaded area represents screening period.

**Figure 6:**
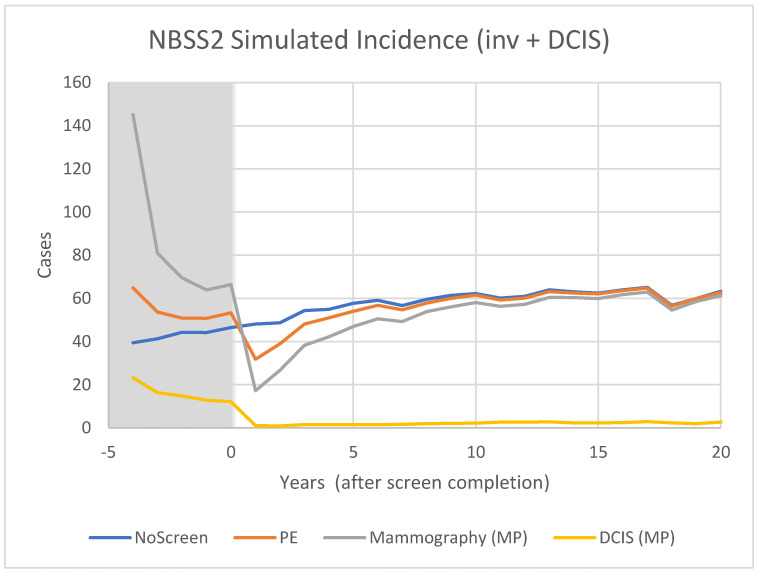
Predicted incidence of breast cancer (OncoSim-Breast model) for women who are unscreened and in the Mammography or Physical Examination arms of the CNBSS2 trial.

**Figure 7:**
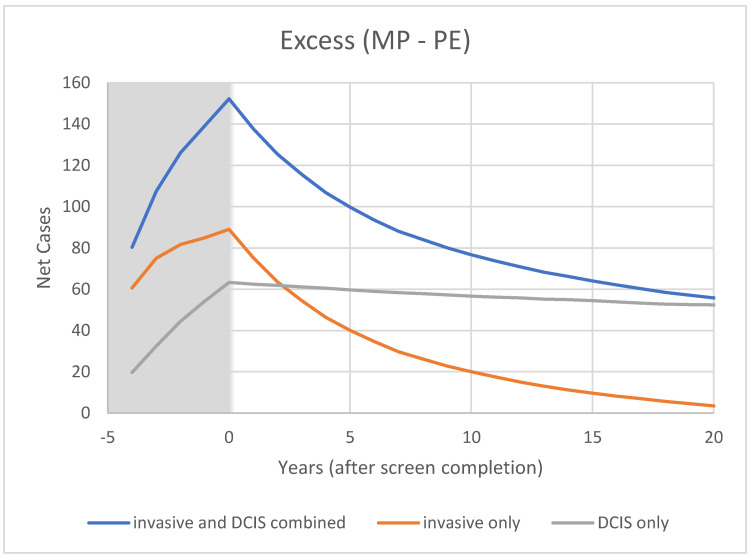
Modeled cumulative excess breast cancer detection in CNBSS2 from OncoSim-Breast.

For CNBSS1 the model breast cancer predictions are shown in Figure 4 for those in the mammography and usual care arms of the trial as well as for unscreened women. Here, annual screening begins at −4 years and Year 0 represents the point when screening ends. In Figure 5, the excess cancers (difference between MP and UC arms) are shown with separate curves for invasive cancers, all cancers and for DCIS only. Figure 6 and Figure 7 show the corresponding predictions for cancer incidence and excess cancers for the older women in CNBSS2.

## 4. Discussion

The rationale underlying effective breast cancer screening is that the screening intervention provides lead time; cancers will be found earlier in the screened group. In the CNBSS trials, this would cause an initial excess of cancers in the MP arms and that excess would persist (a) as long as women in one arm were screened, while in the other they were not, or (b) where the lead time in one trial arm was greater than in the other. Both of these conditions would be expected to occur in CNBSS1 because of the use of only one initial physical examination in the UC arm. The second condition would be in effect in CNBSS2 where less lead time would be expected in the PE arm at each screen as compared to that which received mammography plus clinical examination. At some point, however, it would be expected that the cancer incidence in the control arms would begin to catch up and the excess would diminish. As seen in Figure 3, this is exactly what was observed for invasive cancers in the older women in CNBSS2, but this was not seen in CNBSS1. If the excess cancers in the MP group were due to overdetection during the screening period, it would be expected that the absolute excess should remain constant after the intervention. From the continuing increase in the excess in CNBSS1 with time, we can deduce that factors other than overdetection are at play.

The most obvious explanation is that more women with breast cancer or who were more likely to develop breast cancer were recruited into the MP trial arms. The first factor would explain part of the initial excess in cancers in the MP arms in both trials; the second could contribute to the failure of the UC group in CNBSS1 to “catch up” in the number of invasive cancers over successive years.

Another explanation is there was some causal factors associated with screening in the MP arms that would contribute to more cancers. For example, it has been suggested that the additional radiation exposure from X-rays or the compression of the breast in the mammography might be responsible [27]; however, no credible evidence has emerged to support these hypotheses [28,29,30].

Finally, the thoroughness of cancer ascertainment during the post-screening follow up could be different between the MP and control arms. This possibility was suggested by Baines et al. as a possible contributor to the overestimation of overdetection [21]. This is plausible, especially in CNBSS1, because, by having only a single initial screen, women in the UC group were likely to have much less interaction with the Study than those in the MP arm.

### 4.1. Excess of In Situ Cancers

The CNBSS publications presented data on the number of invasive plus in situ cancers found by screening and otherwise, but did not separately indicate the number of in situ cancers found during the period of screening [23,24]. On the other hand, in their overdiagnosis publication, Baines et al. did present both the total number of invasive cancers and invasive plus in situ cancers in each study arm for CNBSS1 and CNBSS2 at the end of the screening period and for times out to 20 years beyond that [21]. The curves in Figure 3 demonstrate the excess in cancer detection in the MP group over the control group in each trial. The differences between the two curves are also plotted and at Year 0 indicate the excess number of in situ cancers detected in the MP arms during the screening period. This was 33 cancers in CNBSS1 (10% of cancers) and 42 in CNBSS2 (11.1% of cancers).

Following the period of screening intervention, if further screening does not occur, the detection of in situ cancers in both arms should fall dramatically. Under these conditions, it is unlikely that cancers in the control arm corresponding to those in the mammography arm that were detected as in situ cancers (and created that excess) will be detected when they are in situ. If the cancers progress, it is more likely that they will subsequently be detected as invasive cancers during the post-screening follow-up of the control group. If they do not progress to become invasive, it is likely that they will never be detected if the control group remains unscreened after t = 0. In this latter case, they contribute to overdetection.

This may explain why, as seen in Figure 3, the curves for total cancers and for invasive cancers remain essentially parallel; the initial excess in in situ cancers remains constant up to 20 years post-screening. All other factors being equal, this would suggest that many of the excess in situ cancers in the MP arms of CNBSS did not progress to become invasive and that much of the overdetection reported in that study is associated with in situ cancer. In situ cancer is not homogeneous [31] and it is possible that in CNBSS, particularly with concerns due to poor image quality, many of those detected were of the less aggressive phenotypes.

It is interesting that, from Baines et al., by subtracting the number of invasive cancers from the total number of cancers reported, it appears that over the 20 years after the screening intervention, only four and two additional in situ cancers were added in the MP and UC arms, respectively, to those detected during the screening period in CNBSS1. For CNBSS2, only a single in situ cancer was added in each arm over that period. This almost certainly implies that there was no effective post-intervention follow-up of in situ events, even though some degree of screening must have occurred in women in both arms during that time. Possibly cancer registries used for ascertaining cancers arising after t = 0 y simply did not record in situ cancers or possibly the overall ascertainment process had gaps.

### 4.2. Model Predictions

The model predictions for CNBSS1 (Figure 4) demonstrate the expected characteristics of the events in a screening regimen. In this case, the model was configured to simulate a screening intervention in the form of a randomized controlled trial with five annual examinations. The curve for unscreened women serves as a reference; there is a gradual monotonic rise of incidence corresponding mainly to increasing age.

For CNBSS1, at the onset of screening (−4 y), the model predicts a sharp increase in incidence in the arm screened with mammography corresponding to the detection of cancers whose state of development exceeded the threshold for screen detection, but had not yet reached that required to surface symptomatically. Once this “prevalence” screen has occurred, the predicted incidence rates are lower because many of the cancers that had accumulated prior to the onset of screening have been removed from the pool for detection. It might be expected that if there are slow growing or indolent cancers present these would largely be found in that initial prevalence screen along with newer, more aggressive cancers.

A sharp increase compared to no screening is also seen at the single screen of the control (UC) group, but in this case the increase is smaller, under the assumption that mammography was more sensitive than physical examination (higher threshold for detection). While the mammography screened group would include both invasive and in situ cancers, very few in situ cancers would be found in the controls. After the first examination at −4 y, the control group would no longer receive screening, so incidence would fall slightly below the level for unscreened women because some of the cancers had been found earlier due to the lead time provided by an expert clinical examination.

As shown in Figure 5 for CNBSS1, the lead time provided by mammography screening and the greater sensitivity for in situ cancer would be expected to cause an excess in the cumulative cancers detected in the screened group and this would build up gradually over the five screening examinations. After t = 0, the excess of invasive cancers in the MP arm is gradually compensated by a greater number in the control arm. Although half of the excess disappeared at 2.5 y and 75% by 7 y, the excess is not completely cancelled until 23 y, suggesting that there is a broad range of growth rates in these cancers detected by screening. At t = 20 y (25 years post entry), the predicted overdetection is 2% for invasive cancers and 13% for invasive plus in situ cancers. This provides a very different picture as to what happens to the excess cancers after the intervention period than that seen in Figure 3 in the data presented by Baines et al. [21] who estimated overdetection rates of 48% and 53%, respectively, at the same time point.

The predicted mortality reduction for five annual screens at t = 10y is 9% compared to the UC arm. This modest mortality reduction occurs because there were only five screens and the control group received an initial physical exam. Compared to no screening, the model predicts a 16.5% mortality reduction for five screens and 50% reduction for annual screening between ages 50 and 74 [32].

It may be worth making a comment about the effect of the choice of denominator in the calculation. If the number of cancers found in the MP arm (284) had been used rather than the number of screen-detected cancers (213), the overdetection estimate for invasive cancers would have dropped from 48% to 36%.

In CNBSS2 (Figure 6), there is a similar behavior at −4 y in both trial arms, again with a larger increase for the women receiving mammography. Screening continues for four more exams, again building up an excess in the mammography group (Figure 7) that is maximum at the cessation of screening (0 y). This predicted excess is greater than for CNBSS1 despite there being fewer women in the cohort; however, the timing of the decrease in excess cancers is similar to that for CNBSS1.

At t = 20 y (25 years post-entry), the predicted overdetection is 1% for invasive cancers and 16% for invasive plus in situ cancers compared to 5% and 16% from Baines et al. The predicted mortality reduction for five annual screens is 6% compared to the PE arm and 16.5% compared to no screening.

It should be mentioned that, although the model was calibrated against empirical incidence data, it is, of course, not perfect, and one should not overinterpret the numerical predictions. While it provides a mechanistic picture of the elements of screening, there are limited data available to describe the development of DCIS and the rate and degree of its transition to invasive cancer, so that there is considerable uncertainty in modeling the timing associated with the disappearance of the excess created by in situ cancers. Nevertheless, the modeling points to a similar behavior as seen both CNBSS trials in that the excess associated with in situ cancers persists for several decades, suggesting a significant sub-population of nonprogressive in situ cancers.

What are the most plausible explanations for the difference in behavior between the two trials and between the experimental and modeling results for CNBSS1? One possibility is that the women in the MP arm had a higher likelihood of having breast cancer. An anomaly of the CNBSS is that women received a clinical breast examination before they were entered in the register for the trial and, therefore, the suggestion has been put forward that non-randomness could have been introduced at this point, prompted by the findings of the nurse examiner [33]. There is now evidence that this did occur for some of the women, particularly those with advanced cancers at study entry, and this is a credible explanation for the fact that the CNBSS trials were the only RCTs that did not show a mortality benefit associated with routine mammography screening [5]. However, there is a suggestion that the imbalance was not limited to advanced cancers. Because in the prevalence (trial entry) episode of screening in both trials a physical examination of the breast was conducted on women in both trial arms, this provides a point in time where one can compare the number of cancers detectable by palpation between the arms. Although the differences did not reach statistical significance, the authors reported that there were approximately 10% more palpable cancers detected in the MP arm than in the usual care arm of CNBSS1 [23]. In CNBSS2, the excess was 13% [24]. Given that the procedure for detection of palpable cancers was the same in the two study arms and that the same nurses and physicians performed the examinations for both trial arms, such a difference is unexpected, whatever its cause, and would contribute in part to an apparent increase in overdetection.

Another possibility is suggested by the difference in the follow-up in the control groups between CNBSS1 and CNBSS2. In the former, after the initial clinical examination, women in the “Usual Care” arm reported on the incidence of breast cancer primarily through mailed, self-administered questionnaires, while women in the control arm of CNBSS2 received four more annual episodes of clinical examination during the screening epoch and presumably developed a closer relationship and commitment to communicating with study personnel during the subsequent follow-up. A similar situation existed in the study arms of both trials. It is possible that cancer arising after the single screening interaction in the Usual Care arm was under-reported and this would lead to an overestimate of overdetection. Supporting this finding is the observation in the publication by Baines et al. that virtually no DCIS was reported in the UC arm for the 20-year period following the screening intervention [21].

The CNBSS trials were heavily criticized due to the poor quality of the mammography. This is supported by the observation that the mean diameter of cancers in the screened group was only 2 mm smaller than that in the control group [22]. This suggests that the lead time afforded by mammography in CNBSS for actively growing cancers was relatively short. Additionally, while the mean diameter of cancers detected in CNBSS was about 2.1 cm, those cancers that were detected at the point where they were nonpalpable had a mean diameter of 1.4 cm. Therefore, most of the cancers detected by screening in the CNBSS were palpable and as mentioned above, there was a tendency for more of those cancers to be in the MP trial arms. In addition, the nonpalpable cancers most likely to be detected with poor quality mammography would be those that grew more slowly and, therefore, possibly would not reach the point of clinical detectability for many years, explaining the long tail in the curve for excess invasive cancers.

The observation that the excess invasive cancers increased over time after the period of screening intervention casts suspicion on the reliability of the use of data from the CNBSS1 trial for estimating overdetection. Documented problems with fair randomization and the tendency toward more palpable cancers in the MP arms of both trials adds to these concerns [23,24,33]. The one observation from these trials that does merit further consideration is the persistence of the excess that is associated with screen-detected in situ cancer.

### 4.3. Overtreatment

The main harm attributed to overdetection of breast cancer is overtreatment. Overtreatment can be considered to be any treatment whatsoever if the cancer is truly overdetected; however, it can also be unnecessarily harsh or aggressive treatment of slowly progressing cancers.

The greatest concerns regarding the overtreatment of breast cancer have been for in situ disease, which is most commonly treated with breast-conserving surgery and radiation therapy. There are now guidelines for acceptable margins (2 mm) for breast conserving surgery that will reduce re-excision rates [34]. For disease that has a low risk of recurring, the possibility of omitting radiotherapy can be considered [35].

However, it is also crucial that in situ disease not be undertreated. In a SEER review of over 144,000 women diagnosed with DCIS, Giannakeas et al. concluded: “…the risk of dying of breast cancer was increased 3-fold after a diagnosis of DCIS” “(compared to women without a diagnosis of DCIS)” [36].

More recently, it has been shown that a multigene panel based on 12 genes associated with invasive cancer recurrence is predictive of local recurrence after DCIS. Combined with the categorical values of age at diagnosis and tumor diameter, low/medium/high DCIS score can predict a range of 10-year recurrence risks of 6.67 varying from 49% to 7% [37,38]. Further work by the same group in a cohort of 1362 women diagnosed with DCIS in Ontario, Canada, found that the application of the Oncotype DX 21 gene recurrence score was prognostic for invasive local recurrence and risk of breast cancer death [39]. These multigene assays help to predict an individual’s risk of recurrence, which can improve treatment recommendations integrating individual-based risk assessment with individual preferences. Further research is underway to identify biomarkers that predict which women with DCIS would and would not benefit from radiation therapy, thereby reducing overtreatment associated with earlier detection of cancer.

## 5. Conclusions

Screening tends to detect breast cancers when they are smaller and at earlier stage and has a marked effect on the detection of in situ cancer [40]. The detection of some of these cancers, which are very slow growing or indolent, provides little or no benefit and should be considered as overdetection. In some cases, however, the degree of overdetection has been greatly overestimated [3,4,21], or at least the estimates are not on solid ground.

Currently, because there are no reliable and accepted in vivo tests to determine which cancers will be progressive and no way of determining which women will die of other causes before an undetected progressive cancer is found clinically, overdetection is a necessary tradeoff for the substantial mortality and morbidity reduction opportunities provided by screening. Nevertheless, overdetection, and more importantly, overtreatment remain issues that must be addressed, particularly for in situ disease.

At the same time, there are opportunities for the improvement of detecting aggressive cancers through screening methods that are preferentially sensitive to these cancers, such as contrast-enhanced imaging [41], or approaches such as breast tomosynthesis that are intended to make cancers more conspicuous by overcoming tissue superposition effects. In addition, the radiomic analysis of screening images could allow better in vivo characterization to more accurately determine which findings require biopsy [42,43] or are indicative of aggressive disease. For example, Tabar et al. have created subclassifications of mammographic findings previously generically referred to as “DCIS” to distinguish acinar findings, which tend to be lower risk from the more ominous presentations accompanied by linear calcifications in the major lactiferous ducts, likely indicating that neoductgenesis is already occurring [44].

Harms associated with overdetection can also be mitigated by reducing subsequent overdiagnosis, through more definitive pathology tools for analysis and characterization of samples from needle biopsy, thereby reducing the amount of over (and, in some cases, under) treatment [35,37,38,39,45,46,47,48].

While it is tempting to reduce overdetection by limiting screening to those who are perceived to be at greatest risk of developing cancer, often referred to as “personalized screening” [49,50,51,52,53], this does not really target the problem as it is the aggressiveness of the cancer or lack thereof, not the likelihood of incidence that is the main driving factor for overdetection. Personalized screening is an excellent concept and research in this area could be of great value, but until we have risk prediction tools whose negative predictive value is extremely high, this would be a dangerous path to follow as it would result in the missed opportunity for the earlier detection and effective treatment of many potentially lethal cancers.

Finally, women, the general public and health care providers must be clearly, accurately and appropriately informed of the benefits provided by screening as well as the limitations or harms associated with phenomena such as overdetection. This has often been conducted poorly or inaccurately and without any weighting related to quality of life, ascribing inappropriately high harms to false positive screens and overdetection [3]. Others have suggested more useful and positive approaches to communication [54]. To allow informed decision making, and as an indicator of respect for human life, it is critically important that this is performed more effectively in the future.

## Data Availability

All data supporting the results reported in this article can be found in the published work listed in the references. Input parameters and results produced from use of the OncoSim-Breast model can be obtained upon reasonable requests to the authors.
